# Modulation of Primary Afferent Nerve Fiber (Ia) Reciprocal Inhibition Under Voluntary and Electrically Stimulated Muscle Conditions: Within-Subject Study Design

**DOI:** 10.3390/jcm14041178

**Published:** 2025-02-11

**Authors:** Sami S. AlAbdulwahab, Seraj H. Altwerqi, Adnan A. Mubaraki, Maha F. Algabbani

**Affiliations:** 1 Department of Rehabilitation Health Sciences, College of Applied Medical Sciences, King Saud University, Riyadh 11451, Saudi Arabia; 2 Rehabilitation Center of King Abdulaziz Specialist Hospital, Taif 26521, Saudi Arabia; 3 Department of Medicine, Faculty of Medicine, Taif University, Taif 21944, Saudi Arabia

**Keywords:** H-reflex, reciprocal inhibition, soleus H-reflex, primary afferent neuron, agonist–antagonist mechanism, Tibialis anterior

## Abstract

**Background:** Reciprocal inhibition (RI) is a spinal reflex that controls posture and movement. The modulation of spinal RI represented by the H-reflex has been studied, before and after voluntary contraction and electrical nerve stimulation but not during voluntary, electrically induced muscle contraction or a combination of voluntary and electrically induced muscle contractions. This study investigates the effects of the ongoing voluntary isometric contraction, the electrically induced isometric contraction, and the combination of voluntary with electrically induced isometric contraction of the Tibialis Anterior (TA) muscle on spinal RI represented by Soleus H-reflex. **Methods:** Eighteen healthy adults participated. Soleus H-reflex and M-response were measured during four different conditions as follows: (1) at rest, (2) electrically induced isometric contraction of the TA, (3) voluntary isometric contraction of the TA with a 1 kg force, and (4) combined voluntary and electrically induced isometric contraction of the TA with a 1 kg force. **Results:** The ANOVA clearly demonstrated significant differences in Soleus H-reflex amplitude across the four recording conditions (F3,16, 17.28, *p* < 0.001). The amplitude at rest was significantly higher than during electrically induced isometric contraction, voluntary isometric contraction, and the combined contraction conditions (*p* < 0.05). Furthermore, the amplitude recorded during the electrically induced isometric contraction condition significantly surpassed that of voluntary isometric contraction and the combined contraction conditions (*p* < 0.05). Moreover, there was no significant difference between Soleus H-reflex amplitude recorded during voluntary isometric contraction and the combined voluntary isometric contraction and electrically induced isometric contraction (*p* < 0.87). The combined voluntary isometric contraction and electrically induced isometric contraction condition had a higher inhibitory effect on the Soleus H-reflex with no significant differences from voluntary isometric contraction. Moreover, both were significantly better than electrically induced isometric contraction (*p* = 0.05). In terms of Soleus H-reflex latency, there was no significant difference among all four conditions (*p* > 0.05), meaning Soleus H-reflex latency was not influenced by the conditions. **Conclusions:** RI can be best modulated by combining voluntary with electrically induced isometric muscle contractions.

## 1. Introduction

Two opposing sets of muscles control joints, which must work synchronously. Thus, when a muscle spindle is stretched and the stretch reflex is activated, the opposing muscle group must be inhibited to prevent it from working against the resulting contraction of the homonymous muscle. This process is called reciprocal inhibition. The reciprocal inhibition of spinal reflex is a neurological process within the spinal cord that coordinates muscle movements. It involves the simultaneous activation of the motor neurons of an agonist muscle and the inhibition of the motor neurons of its antagonist muscle [1].

An inhibitory interneuron in the spinal cord mediates this inhibition. The Ia afferent of the muscle spindle bifurcates in the spinal cord. One branch innervates the alpha motor neuron that causes the homonymous muscle to contract, producing the behavioral reflex [2]. The other branch innervates the Ia inhibitory interneuron, which innervates the alpha motor neuron that synapses onto the opposing muscle. Because the interneuron is inhibitory, it prevents the opposing alpha motor neuron from firing, thereby reducing the contraction of the opposing muscle. Without this reciprocal inhibition, both groups of muscles might contract simultaneously and work against each other [3].

Spinal reflex activity in humans is commonly evoked externally by mechanical or electrical stimulation (ES); this is assessed by several measures, such as the H-reflex. The H-reflex is an electrically induced reflex comparable to the mechanically induced monosynaptic spinal stretch reflex. It assesses alpha motoneuron excitability when intrinsic alpha motoneuron excitability and presynaptic inhibition remain constant. H-reflex triggers the monosynaptic motor units recruited by the afferent (Ia sensory nerve fiber) pathway [4]. It has been used to assess spinal reciprocal inhibition (RI) modulation [5].

Many attempts have been made to manage and modulate spinal RI and co-contraction to facilitate physical activity in patients with neurological conditions. One of these attempts was using ES to activate spinal RI via stimulating afferent Ia fibers [6,7,8].

Spinal RI has been reported to be lower during the co-contraction of the TA and Soleus muscles than at rest in healthy subjects [9]. It was also stated that spinal RI depended on plantarflexion strength and diminished as Soleus muscle strength increased [9]. Moreover, abnormal irregular excessive co-contraction was assumed to be a disorder in spinal RI [10]. In repetitive and quick exercises, excessive co-contraction inhibits smooth joint movement and causes agonist muscle fatigue [11]. The excessive co-contraction among antagonist muscles decreases exercise performance, requiring agility in athletes [12]. Pathologically, such abnormal excessive co-contraction was reported in many cases with upper motor neuron disorders and manifested as spasticity or rigidity in patients with spastic diseases, cerebellar ataxia, Parkinson’s disease, stroke, and spinal cord injury [13,14,15]. Moreover, co-contraction increases in older adults, particularly in lower limbs, impairing gait function and raising the risk of falling [16,17]. Excessive co-contraction between agonist–antagonist muscles was also reported to decrease joint mobility [18,19]. Thus, humans need normal modulation of RI for safe and effective physical activity.

ES is widely used in treating muscle disuse atrophy and neuromuscular re-education; it can generate controlled limb movements in patients with upper motor neuron lesions [20,21,22]. The effectiveness of ES in controlling limb movement in patients with neurological problems has been related to a reduction in spasticity, the activation of weakened muscles, and an improvement in spinal reflexes [20,21,22].

In this context, the literature reviews showed further diverse reports about RI modulation. Al-Abdulwahab and Al-Khatrawi (2009) reported that the visible and minimal continuous electrically induced isometric contraction of hip abductors during walking significantly improves gait and reduces spasticity in the hip adductor muscles of children with spastic diplegia cerebral palsy (CP) [23]. They related such improvement to the proper activation of RI during waking. Sabut (2010) reported that ES to TA during walking in patients with stroke significantly improves walking, range of motion, and spasticity [24,25]. Furthermore, Al-Abdulwahab (2011) stated that the simultaneous continuous ES of both hip abductors at a motor level and hip adductors at a sensory level of children with spastic diplegia CP during walking significantly improves gait performance, muscle tone, and knee position [26]. He related such improvement to a more normalized activation of hip abductor–adductor muscle RI and the additional facilitation of more muscle fibers in the hip abductor. Warshaw et al. (2021) stated that ES to the Gluteus Medius during walking significantly improves the gait, balance, and functional independence of patients with hemiparesis stroke [27]. These studies indicate that the ongoing ES of agonist muscles might induce a positive modulation of spinal RI to manage spasticity in the antagonist’s muscle. Thus, further studies on this issue were needed to improve physical rehabilitation programs.

Moreover, AlAmer et al. (2020) investigated Soleus H-reflex modulation during erect, slumped, and slouched postures [28]. They reported that the Soleus H-reflex modulated with different postural positions and confirmed the reliability of using Soleus H-reflex in assessing spinal modulation. This could indicate the adaptability of spinal reflexes, as represented by the Soleus H-reflex, in various challenging physical conditions and the appropriate use of H-reflex to assess motor control and coordination [28]. Further, Qin et al. (2021) reported that in healthy subjects, Soleus H-reflex significantly decreased in the standing position compared to the prone position [29]. In contrast, Soleus H-reflex in patients with spastic stroke increased in the prone and standing positions on both sides. Such modulation was related to reciprocal inhibition at the spinal level and connected to an overall upregulation of the anterior horn cell’s excitability.

Furthermore, Sidney et al. (2023) demonstrated a decrease in Soleus H-reflex during virtual reality stimulation on standing and suggested that virtual reality stimulation modulates spinal reflex [30]. Aldhaferi (2024) found that the ES of simultaneous ongoing, visible isometric contraction to Gluteus Medius and TA muscles significantly improves sit-to-stand performance and walking parameters, and it reduces spasticity in the hip adductor and calf muscles in patients with stroke. He related such improvements to a possible increase in the motor unit activation of the hip abductor and TA muscles and reciprocal inhibition normalization [31].

In another aspect, it is already known that muscle types are divided into slow-twitch tonic fibers (Type I fibers) and fast-twitch phasic fibers (type II fibers). During voluntary contraction, the muscle fibers are progressively recruited in an orderly manner from small to large muscle fibers according to the intensity of the contraction, mainly from the depth of the muscle to the surface, since small fibers (slow-twitch fibers, tonic fibers, Type I fibers) are primarily located deeper in the muscle [3]. In contrast, during ES contraction, the muscle fibers are recruited from large to small according to the intensity of ES, mainly from the surface muscle to the deep muscles, since large fibers (fast-twitch, phasic fibers, or type II fibers) are primarily located on the surface of the body [3,32]. Thus, the higher the ES intensity, the greater the recruitment of deeper and superficial fibers. The lower the ES intensity, the more surface fibers are recruited [3].

Therefore, this study investigated how different types of isometric contractions (voluntary, electrically induced, and combined) affect human spinal reflexes. This information is clinically important for developing effective physical rehabilitation programs that instantly activate reciprocal inhibition to facilitate mobility and physical activities in patients with upper motor lesions. Such programs may replace conventional programs that require time and effort and are costly.

## 2. Methods

### 2.1. Study Design and Sitting

An observational repeated-measure within-subject study design was employed. Data were collected in the rehabilitation department in Your Doctor Consultations polyclinics in Al-Taif, Saudi Arabia, between May and October 2023.

### 2.2. Participants

Healthy adults aged 20–40 years with no history of neurological or musculoskeletal conditions voluntarily participated in the study. Participants with a body mass index (BMI) above 30, lower limb injuries within six months, or those who could not complete the tests were excluded. Participants were recruited from the rehabilitation department’s therapists using flyers.

### 2.3. Sample Size Estimation

The sample size estimation was determined using G-power with a medium effect size of 0.3, an alpha level of 0.05, and a power of 0.80. The required sample size was *n* = 17.

### 2.4. Ethical Consideration and Informed Consent

The study was approved by the Institutional Review Board of King Saud University, College of Medicine (KSU-IRB Approval 23 January 2023 Ref. No. 23/0008/IRB-A). The participants gave their written consent to participate in the study.

### 2.5. Outcome Measures and Materials

An electromyogram (EMG) device (UltraPro^®^ S100 EMG/NCS/EP neurodiagnostic system, Galway, Ireland) was used to detect the modulation in the spinal Ia RI by recording Peak-to-Peak Soleus H-reflex and M-wave amplitudes. The Soleus H-reflex amplitude is a valid indicator of the Ia excitatory effect on the target motoneurons [33].

A small portable, two-channel, and rechargeable battery-powered Electrical Stimulation Unit manufactured by MEDIHIGHTEC MEDICAL Co., Ltd., Taipei City, Taiwan, edition: V2.0, with a supply voltage of 9 V, was used to induce the electrical stimulation. It had biphasic asymmetrical waveforms, with a frequency of 35 Hz, a pulse width of 80 μs, and an intensity causing visible and isometric contraction. The intensity of the stimulator was delivered into TA muscle via two self-adhesive electrodes; each electrode was square and (3 cm × 3 cm) in size.

A handheld dynamometer (HHD), the Nicholas Manual Muscle Tester (model 01160, Lafayette Instrument Company, Lafayette, IN, USA), was used to measure the maximum force required to break an isometric contraction. This method has been proven reliable for assessing lower limb muscle strength in adults.

### 2.6. Procedures

The maximum Soleus H-reflex with minimal M-response was measured on the dominant side in four different conditions during a single session as follows: (1) At rest, with the ankle slightly flexed (10 degrees). (2) During the ongoing, visible electrical stimulation of the TA muscle, with the ankle maintained at 10 degrees. (3) During the voluntary contraction of the TA muscle with a force of 1 kg while keeping the ankle at 10 degrees. (4) During the combined voluntary and electrically induced contraction of the TA muscle with a total force of 1 kg while maintaining the ankle at 10 degrees.

The participants lay face down on the table with a small pillow under their chest and arms under their forehead. Their head was positioned neutrally, and their feet were placed comfortably over the table’s edge. The skin on the back of their knee and the posterior of their legs was cleaned with alcohol.

Then, the Soleus H-reflex and M-wave stimulation and recording procedures were carried out similarly to those of David Burk (2016) [34]. The Soleus H-reflex/M-response recording signals were amplified by 500–2000 × using differential amplification, filtered at a 20–10,000 Hz bandwidth, digitized, and stored on a computer for analysis. A complete and straightforward explanation of the procedure was given to ensure the controlled reaction from the participant, including a description of the sensations and responses produced [34].

**Figure 1:**
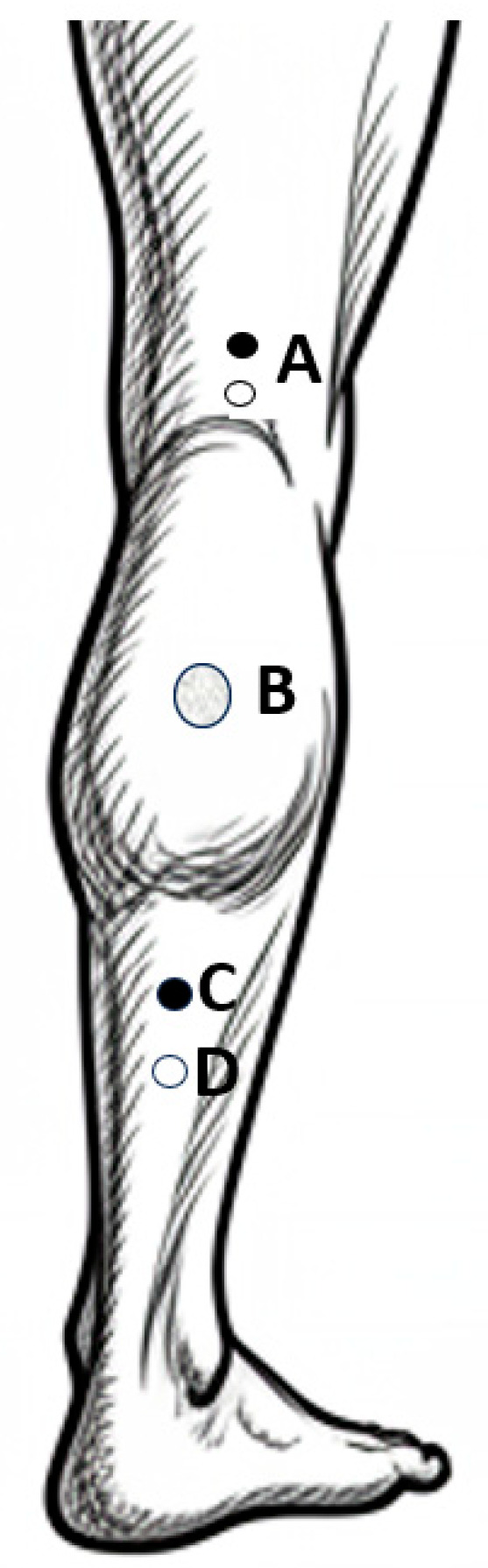
The EMG electrode placement. A: pin electrode position; B: ground electrode; C: active electrode; D: reference electrodes.

The Soleus H-reflex/M-response stimulation-recording procedure and parameters were as follows:

Two recording adhesive surface single electrodes (active and reference electrodes) were positioned over the Soleus muscle with the active electrode (1 cm^2^) proximal to the reference electrode (1 cm^2^) at 2 cm distal to the bifurcation of the gastrocnemii and in line with the Achilles tendon. A ground surface adhesive electrode (1 cm^2^) was positioned midway between the stimulation and recording electrodes on the calf’s skin (Figure 1).

Then, a handheld stimulator silver-silver chloride pin electrode was placed, with coupling gel, longitudinally on the tibial nerve in the popliteal fossa, providing a probe that mounts the cathode and anode at a fixed distance of 2 cm apart. The intensity control located in the insulated handle, though bulky, simplifies the operation for a single examiner (Figure 1).

The stimulation parameters were started at an intensity of 1.0 mV and a 1.0 ms pulse duration and increased slowly and gradually, eliciting the maximum Soleus H-reflex and minimum M-response. Two-minute practice trials of the elicited and recorded H-reflex were carried out to familiarize the participants with the H-reflex stimulation and recordings to reach the maximum response of Soleus H-reflex with minimum M-response. Then, the H-reflex stimulation was recorded under the following conditions.

Condition 1 (baseline measurements of the M-response and H-reflex of Soleus muscle), after reaching the maximum H-reflex, the intensity of the H-stimulation was fixed in all conditions. At least five readings of the maximum Soleus H-reflex with the minimum stable M-response were recorded in each condition to represent spinal RI modulation. There were at least five seconds of rest between each recording. Also, there was a rest of at least two minutes between each condition to avoid muscle fatigue. The mean amplitude of the five readings of the maximum Soleus H-reflex and M-response were calculated.

Under condition 2 (electrically induced visible isometric contraction of the TA muscle), the intensity of ES was fixed at the point of the visible isometric contraction of the TA muscle. It was expected that ES caused constant artifacts that interfered with the H-reflex recordings. To overcome this issue, the H-reflex and M-response were recorded several times to avoid overlapping with ES artifacts. Thus, the H-reflex and M-response were recorded between the ES artifacts.

Under condition 3 (voluntarily isometric contraction of the TA muscle), the participants were asked to contract the TA muscle voluntarily, resisting ankle movement with the handheld dynamometer to obtain a 1 kg force. The ankle was maintained in approximately 10-degree plantarflexion.

Under condition 4 (electrically induced TA muscle contraction combined with the voluntary isometric contraction of the TA muscle), the intensity of ES was increased and fixed at the point of visible isometric contraction of the TA muscle. At the same time, the participant was asked to contract the TA muscle voluntarily, resisting the movement of the ankle with the handheld dynamometer to obtain a 1 kg force. The ankle was maintained in approximately 10-degree plantarflexion during the recording process. It was also expected that ES caused constant artifacts that interfered with the H-reflex recordings. To overcome this issue, the H-reflex and M-response were recorded several times to avoid overlapping with ES artifacts. Thus, the H-reflex and M-response were recorded between the ES artifacts.

The electrically induced visible isometric contraction of the TA muscle was started by cleaning and moistening the skin over the TA with alcohol swabs to reduce possible skin resistance and irritation. Then, a self-adhesive negative electrode (3 × 3 cm) was attached over the motor point and belly of the TA muscle. A positive electrode (3 × 3 cm) was placed 3 cm below the negative electrode in the longitudinal axis.

The electrodes were connected to the ES unit. Then, ES intensity was gradually increased until an isometric contraction was visually observed. This intensity and level of contraction were maintained throughout conditions 2 and 4.

### 2.7. Statistical Analysis

Data manipulation for the current study was performed using IBM SPSS (IBM SPSS Statistics for Windows, Version 25.0. Armonk, NY, USA: IBM Corp). The mean and standard deviation were calculated to show the descriptive statistics of the continuous variables. The Shapiro–Wilk test was used to test the data distribution normality. A one-way repeated measure ANOVA was used to compare the means across the study’s repeated observations, and the statistical significance level was alpha 0.05.

## 3. Results

### 3.1. Demographic Data

Eighteen healthy subjects participated in the present study, with a mean age of 29.9 ± 4.8 years, height 171 ± 0.06 cm, weight 74.22 ± 12.52 kg, and body mass index (BMI) 25.18 ± 3.03 kg/cm^2^ (Table 1).

### 3.2. The Soleus H-Reflex Peak-to-Peak Amplitude and Mean Latency Under the Different Conditions

The Soleus H-reflex amplitudes and mean latency in all conditions were normally distributed (*p* > 0.05). The mean amplitude and latency of the Soleus H-reflex are described in (Table 2).

The ANOVA revealed significant differences in the Soleus H-reflex amplitude across the four conditions (F_3,16_, 17.28, *p* < 0.001).

The Soleus H-reflex at rest (Condition 1) exhibited a significantly higher amplitude when compared with its amplitude during electrically induced isometric contraction, voluntary isometric contraction, and combined voluntary isometric contraction along with electrically induced isometric contraction (*p* < 0.05). Additionally, the Soleus H-reflex amplitude recorded during the electrically induced isometric contraction was significantly higher than that recorded during voluntary isometric contraction and combined contraction (*p* < 0.05).

Moreover, there was no significant difference between the Soleus H-reflex amplitude recorded during voluntary isometric contraction and the combined voluntary isometric contraction and electrically induced isometric contraction (*p* < 0.87). (Table 3)

There was no significant difference in Soleus H-reflex latency between all four conditions (*p* > 0.05), meaning the mean Soleus H-reflex latency was the same and was not influenced by the different conditions (Table 4).

**Figure 2:**
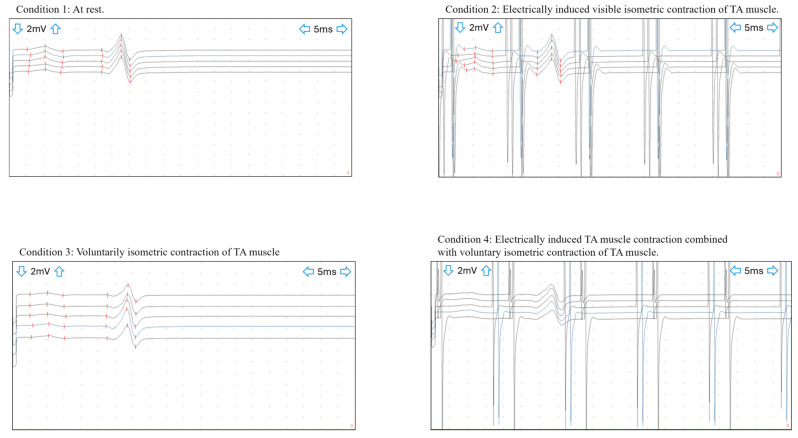
Soleus M-response and H-reflex amplitudes (mV) and latencies (ms) during the four different conditions. These conditions exhibited an inhibitory effect, reducing the H-reflex amplitudes.

The Soleus H-reflex and M-response recordings during the four conditions are clearly shown in Figure 2. The Soleus H-reflex amplitudes were gradually and continually reduced during the Conditions. The first condition was recorded to establish a baseline for the M-response and Soleus H-reflex. The baseline (condition 1) showed a relatively minimal M-response with the maximum H-reflex. The second, third, and fourth conditions showed a comparably stable M-response to the baseline but an obvious gradual reduction in Soleus H-reflex amplitudes. In conditions 2 and 4, a fixed shape of ES artifacts appeared between the M-response and Soleus H-reflex amplitude waves.

## 4. Discussion

**Scheme 1:**
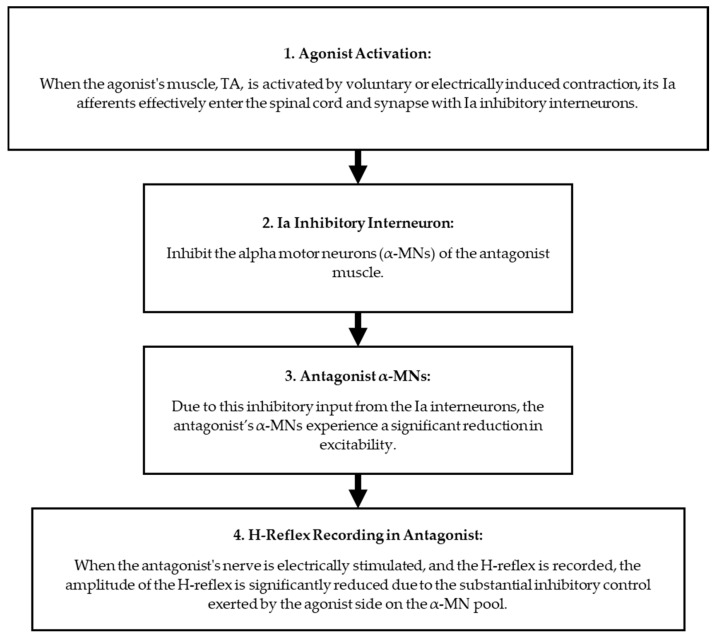
Ways in which RI can influence the H-reflex amplitude in an antagonist muscle.

This study showed that the Soleus H-reflex amplitudes could be modulated by the voluntary and electrically induced contraction of antagonist muscles. The separate voluntary isometric contraction, electrically induced visible isometric contraction, and a combination of both voluntary isometric and electrically induced contractions of the TA muscles inhibited Soleus H-reflex amplitudes in healthy subjects (Scheme 1). Moreover, combining voluntary isometric contraction with electrically induced contraction inhibited Soleus H-reflex amplitudes more than the separate isometric voluntary contraction or electrically induced visible contraction of TA muscles.

Thus, it can be proposed that a combination of voluntary isometric contraction with electrically induced contraction, the separate voluntary isometric contraction, and the separate electrically induced contraction of the antagonist muscles could help enhance the modulation of spinal reflexes, particularly RI, to improve the physical performance of patients with upper motor neurological diseases. Aldhaferi (2024) reported that during the continuous electrically induced visible isometric contraction of TA muscle, spasticity in calf muscles is instantly reduced with an improvement in the gait parameters of stroke patients [31]. He related this improvement to the facilitation of the RI spinal reflex and the activation of type II muscle fibers in the TA muscle.

H-reflex is commonly used to study spinal reflexes, spinal motoneuron units, and movement control mechanisms by estimating the reflex excitability of several muscles. H-reflex modulations are usually associated with performing various motor conditions [35]. Active ankle planter flexion facilitates the Soleus H-reflex, whereas active ankle dorsiflexion inhibits the Soleus H-reflex amplitude [36]. It is well known that the H-reflex peak-to-peak amplitude indicates the number of motor units recruited during a given external stimulus intensity. Moreover, a reduction or inhibition in the H-reflex amplitude implies a decrease in the excitability of the reflex pathway and recruited number of motor units. The H-reflex amplitude has been used to investigate various spinal reflex mechanisms that affect motoneuron excitability and pathways, such as reciprocal inhibition reflex [35]. In the present study, the reduction in the Soleus H-reflex peak-to-peak amplitude during the testing conditions further supports using H-reflex to study spinal reciprocal inhibition to improve physical rehabilitation programs for patients with stroke.

It has been reported that there is a strong correlation between the strength of the ankle dorsiflexion and the degree of the inhibition of the Soleus H-reflex amplitude. The inhibition of the Soleus H-reflex amplitude increases as the voluntary ankle dorsiflexion contraction strengthens [37,38]. Moreover, it is further confirmed that voluntary isometric ankle dorsiflexion reduces Soleus H-reflex motoneuron excitability due to reciprocal inhibition modulation [39]. The degree of the reciprocal inhibition of Soleus H-reflex motoneurons and the increase in TA strength activity are linearly related [38]. In the same context, the modulation of Soleus H-reflex amplitude during active ankle dorsiflexion and active ankle plantarflexion was linked with the following two mechanisms: the reciprocal inhibition between agonist–antagonist muscle contraction during active ankle dorsiflexion that evoked reciprocal Ia inhibition on the triceps surae muscle and the increase in the excitability of alpha motoneurons of the soleus muscle during active ankle plantarflexion. Such reciprocal inhibition modulation could explain the reduction in the Soleus H-reflex amplitude during the voluntary, electrically induced, and the combination of voluntary and induced isometric TA muscle dorsiflexion in the present study.

Furthermore, the present study’s gradual reduction in Soleus H-reflex amplitude during induced, voluntary, and a combination of voluntary and induced isometric ankle dorsiflexion could be due to the activation of more reciprocal Ia inhibition on the TA muscle. A gradual increase in ankle dorsiflexion strength causes a gradual reduction in Soleus H-reflex [36,37]. This could imply that the amplitude of Soleus H-reflex can be modulated and reduced by the degree of strength of voluntary or electrically induced isometric ankle dorsiflexion. Moreover, the ES was reported to generate a synchronized fixed [40], non-selective [41], and mostly partial motor unit recruitment [32]. The ES-induced motor unit recruitment mainly involves the high-threshold motor units of type II fast-twitch fibers, mostly located in the superficial areas of the skeletal muscles [42]. It is documented that the recruitment of motor units is asynchronous during voluntary muscle contraction while synchronous during ES [41]. It is also known that motor units are recruited sequentially to generate a given level of voluntary muscle contraction. The motor units are recruited in order, from smallest (slow twitch-type I muscle fibers) to largest (fast twitch-type II muscle fibers), as voluntary contraction increases. In a voluntary manner, type I muscle fibers with low activation thresholds are recruited first. Type II muscle fibers are engaged if they cannot generate the required strength for the specific activity [43]. In contrast, ES reverses the activation order [44].

Thus, the difference in motor unit recruitment between voluntary and electrically induced contraction may explain the different levels of H-reflex amplitude inhibition reported in the present study. It also indicates the possibility of different pathways to initiate the RI reflex, that is, the slow twitch-type I muscle fibers activation pathway and the fast twitch-type II muscle fibers activation pathway. Furthermore, combining voluntary with electrically induced contraction may simultaneously activate both slow twitch-type I muscle fibers and fast twitch-type II muscle fibers in the TA muscle, further inhibiting the Soleus H-reflex amplitude. Notably, the voluntary isometric contraction used in the present study produced approximately 1 kg of dorsiflexion force to primarily activate the slow twitch-type I muscle fibers. Likewise, the electrically induced contraction used in the present study produced just visible isometric dorsiflexion to primarily activate the fast twitch-type II muscle fibers.

Consequently, the clinical implication of such results is that voluntary, electrically induced, and a combination of voluntary and electrically induced contraction may be appropriate to instantly activate reciprocal inhibition to facilitate mobility and physical activities in patients with upper motor lesions. Conventionally, physical therapists use Bobath, Brunnstrom, Proprioceptor Neuromuscular Facilitation, stretching, and weight-bearing exercises to reduce spasticity and facilitate mobility and functional activities. Although these exercises are effective, they require time and effort, and they are costly. Thus, the electrically induced activation of a nondominant spastic group of muscles facilitates reciprocal inhibition, reducing spasticity in the dominant antagonist spastic group of muscles. The continuous visible isometric and electrically induced contraction of the ankle dorsiflexor and hip abductor muscles reduces spasticity in the calf and hip adductor muscles and improves the sit-to-stand and walking parameters in stroke patients [31].

In the present study, the M-response was almost unchanged throughout the Soleus H-reflex recordings to ensure a similar stimulus intensity was used across the four testing conditions. Thus, any change in the Soleus H-reflex amplitude would represent a genuine alteration in the motoneuron pool that innervates the Soleus muscle and reciprocal inhibition reflex.

Moreover, some might argue that the inhibition in the Soleus H-reflex amplitude is due to an artifact from the ES device used to activate the TA muscle. The artifact disturbance in EMG studies from surrounding electrical machines is a given if it has not been dealt with properly. However, the artifact waves are distinct and consistent in shape, duration, and frequency [45]. In the present study, extreme care was taken to ensure that the Soleus H-reflex was recorded between the artifact waves.

Moreover, reducing Soleus H-reflex amplitude under conditions 2 and 4 confirmed that there was no overlap between artifact waves and Soleus H-reflex. The overlap between both waves could cause deformities or hidden H-reflexes, and small artifact waves could cause an increase in H-reflex amplitudes. Thus, the recorded Soleus H-reflex during the four conditions represents a true IR spinal modulation.

Furthermore, the present study’s Soleus H-reflex amplitude and latency values were almost similar to the previously published values [23,30]. This could indicate that the Soleus H-reflex stimulation-recording procedures used in the present study were consistent with other studies.

This study has several limitations. The study sample was small, including only healthy subjects with a limited age range; however, the G*Power determined that a total of seventeen subjects is sufficient to achieve a statistical power of 0.80 for detecting the desired effect size. The repeated-measures design and normally distributed data employed in this study can achieve power levels similar to larger between-subject samples due to lower within-subject variability. Additionally, ethical and practical constraints often make it difficult to recruit larger samples. A small sample size is reported to be valid if the power analysis confirms that it is adequate for the expected effect size. It has been reported that a small sample (e.g., 17) can be perfectly valid when a careful power analysis confirms it is adequate for the expected effect size under a repeated-measures design with normally distributed data [46,47,48]. Furthermore, a larger sample size with different and older age groups of healthy individuals with normal aging-related neuromuscular changes and patients with neurological conditions exhibiting pathological neuromuscular alterations and variations in spinal excitability should be studied and included in future studies. Moreover, the H-reflex stimulation and recording electrodes were pin and single-surface, respectively. Bar surface electrodes may be more convenient for such a study.

## 5. Conclusions

RI represented by the H-reflex can be instantly modulated with voluntary isometric muscle contraction and electrically induced muscle contraction, and it can be combined with voluntary and electrically induced muscle contraction. Furthermore, the voluntary and electrically induced isometric muscle contractions cause different degrees of inhibitory RI modulation. This could be related to the type of muscle fiber activation during voluntary and electrically induced muscle contraction.

## Figures and Tables

**Table 1:** Descriptive statistics for the demographic data (*n* = 18).

	Mean	Standard Deviation
Age (years)	29.89	4.81
Height (centimeters)	171	0.06
Weight (kilograms)	74.22	12.52
BMI (kilogram/centimeter ^2^)	25.18	3.03

**Table 2:** Descriptive statistics of the Soleus H-reflex peak-to-peak amplitude and mean latency during the different conditions.

	Soleus H-Reflex Amplitude (mV)		Soleus H-Reflex Latency (ms)	
	Mean	Std. Deviation	Mean	Std. Deviation
Condition 1	3.92	1.55	29.27	1.63
Condition 2	3.28	1.76	29.36	1.45
Condition 3	2.16	1.32	29.47	1.42
Condition 4	1.80	0.82	29.47	1.42

Condition 1 = at rest; condition 2 = electrically induced visible isometric contraction of the Tibialis anterior muscle; condition 3 = voluntarily isometric contraction of the Tibialis anterior muscle; condition 4 = electrically induced Tibialis anterior muscle contraction combined with the voluntary isometric contraction of the Tibialis anterior muscle.

**Table 3:** Bonferroni pairwise comparisons for the four conditions of the Soleus H-reflex amplitude in mV.

H-Reflex Measurements	H-Reflex Measurements	Mean Difference	Sig.	95% Confidence Interval for Difference	
				Lower Bound	Upper Bound
Condition 1	Condition 2	0.64 *	0.01	0.11	1.16
	Condition 3	1.75 *	0.00	0.85	2.66
	Condition 4	2.11 *	0.00	1.15	3.08
Condition 2	Condition 3	1.11 *	0.00	0.34	1.88
	Condition 4	1.47 *	0.00	0.56	2.38
Condition 3	Condition 4	0.36	0.87	−0.34	1.08

Condition 1 = at rest; condition 2 = electrically induced visible isometric contraction of the Tibialis anterior muscle; condition 3 = voluntarily isometric contraction of the Tibialis anterior muscle; condition 4 = electrically induced Tibialis anterior muscle contraction combined with the voluntary isometric contraction of the Tibialis anterior muscle. * Significant at *p* < 0.05.

**Table 4:** Bonferroni pairwise comparisons between the Soleus H-reflex latency recorded under the different conditions.

H-Reflex Latency	H-Reflex Latency	Mean Difference	Sig. *	95% Confidence Interval for Difference	
				Lower Bound	Upper Bound
Condition 1	Condition 2	−0.08	1.00	−0.43	0.26
	Condition 3	−0.19	0.54	−0.52	0.13
	Condition 4	−0.19	0.54	−0.52	0.13
Condition 2	Condition 3	−0.11	0.97	−0.34	0.12
	Condition 4	−0.11	0.98	−0.34	0.12
Condition 3	Condition 4	−0.11	0.99	−0.34	0.12

Condition 1 = at rest; condition 2 = electrically induced visible isometric contraction of the Tibialis anterior muscle; condition 3 = voluntarily isometric contraction of the Tibialis anterior muscle; condition 4 = electrically induced Tibialis anterior muscle contraction combined with the voluntary isometric contraction of theTibialis anterior muscle. * Significant at *p* < 0.05.

## Data Availability

The data that support the findings of this study are available upon request from the corresponding author. The data are not publicly available due to their containing information that could compromise the privacy of the participants.
